# The relationship between college students’ legal cognition and maladaptive risk-taking behaviors: the moderating effect of need for cognitive closure

**DOI:** 10.3389/fpsyg.2025.1717060

**Published:** 2025-11-26

**Authors:** Yu Hu, Qiang Ding, Hui Liu, Shuhui Xu

**Affiliations:** 1 Department of Psychology, Wenzhou University, Wenzhou, Zhejiang, China; 2 Center for Psychology and Behavior Research, Wenzhou University, Wenzhou, Zhejiang, China; 3 College of International Education, Wenzhou University, Wenzhou, Zhejiang, China; 4 School of Foreign Languages, Northwest University, Xi’an, Shaanxi, China

**Keywords:** legal cognition, concrete legal cognition, abstract legal cognition, need for cognitive closure, need for structure, decisiveness

## Abstract

Legal socialization refers to the developmental process through which individuals form beliefs, values, and attitudes toward the law. A key component of this process is legal cognition, which includes concrete legal cognition and abstract legal cognition. While prior research links legal cognition to reduced antisocial behavior, its role in maladaptive risk-taking remains unclear. Additionally, how the two dimensions of Need for Cognitive Closure (NFC)—need for structure and decisiveness—moderate these effects is not well understood. In this study, 396 college students completed measures of legal cognition, maladaptive risk-taking, and NFC. Results showed that both concrete and abstract legal cognition negatively predicted maladaptive risk-taking. Need for structure strengthened these negative associations, while decisiveness weakened the link between concrete legal cognition and risk-taking but had no significant effect on abstract legal cognition. These findings suggest that the two types of legal cognition play distinct protective roles, and that the two dimensions of NFC differently shape how legal beliefs influence risk-taking behavior, providing guidance for teachers and school administrators in designing interventions to reduce maladaptive risk-taking among students.

## 1 Introduction

Adolescence is characterized by heightened engagement in maladaptive risk-taking behaviors, such as substance use, reckless driving, and delinquency, which existing frameworks only partially explain (Willoughby et al., 2021; Leather, 2009). Such behaviors are associated with adverse developmental outcomes, including academic underachievement, mental health problems, and future antisocial tendencies (McLeod et al., 2012). The dual systems model attributes this vulnerability to asynchronous neural maturation: the socio-emotional system, linked to reward sensitivity and affective reactivity, develops earlier and faster than the cognitive control system, which continues to mature into the mid-20s (Shulman et al., 2016; Steinberg, 2008). Social bonding theory highlights that strong attachments to prosocial figures (e.g., parents, teachers) serve as protective factors by promoting adherence to societal norms, whereas differential association theory emphasizes that peer networks can transmit deviant norms, increasing susceptibility to maladaptive behaviors (Alduraywish, 2021; Costello and Laub, 2020; Hirschi, 1969; Wang et al., 2018). Integrating these perspectives underscores the importance of both social context and individual cognition in shaping adolescent risk-taking, with legal cognition—an index of legal socialization and internalized norms—serving to bridge social-bonding and differential-association frameworks.

### 1.1 Legal cognition and risk-taking behaviors

Risk-taking refers to choices favoring immediate rewards despite potential harm (Ben-Zur, 2009), with maladaptive forms undermining individual well-being (Hansen and Breivik, 2001; Weigard et al., 2014). We argue that legal cognition—mental representations of law—functions as a proximal protective factor. Abstract legal cognition (beliefs about law’s purposes, values, and societal functions) fosters principled internalization and constrains antisocial intent (Tyler, 2006; Slocum et al., 2016; Xu and Yan, 2022). Concrete legal cognition (knowledge of statutes, rights, and sanctions) clarifies behavioral contingencies and promotes deliberative deterrence (Xu and Yan, 2022; Slocum et al., 2016).

Existing empirical evidence supports these distinctions: positive legal emotions can attenuate the influence of dispositional tendencies—such as sensation seeking—on risk-taking behaviors (Wang et al., 2025), and in the domain of Internet addiction, belief in a just world is associated with lower addiction levels particularly when accompanied by higher degrees of abstract legal cognition (He et al., 2025). Despite these findings, systematic understanding of how abstract versus concrete legal cognition develop across life stages remains limited. Developmental psychology suggests that as cognitive and moral reasoning mature, individuals’ comprehension of abstract principles (e.g., justice and the purposes of law) advances, shaping judgments and behavioral choices in complex or novel situations (Piaget, 1932; Kohlberg, 1981; Reyna and Farley, 2006). This gap justifies the present study’s focus on differentiating these dimensions of legal cognition.

The practical significance of distinguishing abstract and concrete legal cognition lies in their differential applicability: abstract cognition guides principled reasoning across diverse contexts, while concrete cognition informs context-specific behavior and understanding of legal consequences (Xu and Yan, 2022). Clarifying their unique contributions can help tailor interventions to strengthen legal understanding and reduce risk-taking in adolescents and young adults.

Accordingly, we hypothesize:

*H*1: Both abstract and concrete legal cognition will be negatively associated with maladaptive risk-taking behaviors.

### 1.2 The moderating role of cognitive closure

Need for Cognitive Closure (NFC) reflects the motivational preference for rapid and stable resolution of ambiguity and comprises two distinct dimensions: need for structure (seizing) and decisiveness (freezing; Webster and Kruglanski, 1994; Liu et al., 2018; Nagy et al., 2018). Seizing reflects urgency in constructing coherent schemas and intolerance of ambiguity; freezing reflects rigidity in maintaining initial judgments.

Research on NFC and risk-taking is mixed: when NFC primarily motivates avoidance of uncertainty, high-NFC individuals may adopt precautionary, risk-averse strategies (Schumpe et al., 2017). Conversely, the urgency facet of NFC—propensity for rapid conclusions—can facilitate impulsive or risk-seeking decisions in some contexts (Andrews, 2013; Kruglanski and Webster, 1996). Disaggregating NFC into seizing and freezing clarifies these mechanisms and their interaction with legal cognition.

Mechanistically, seizing accelerates integration of abstract concepts (e.g., justice, fairness) into decision frameworks, strengthening the behavioral impact of abstract legal cognition in novel risk contexts (Kruglanski et al., 1993; Roets and Van Hiel, 2011; Thompson et al., 2001). By contrast, freezing promotes reliance on established heuristics, making concrete legal knowledge effective in familiar contexts but less adaptive under novel or shifting circumstances (Webster and Kruglanski, 1994; Roets and Van Hiel, 2011). Accordingly:

*H*2a: The need-for-structure dimension will strengthen the negative association between legal cognition and maladaptive risk-taking.

*H*2b: The decisiveness dimension will weaken the negative association between legal cognition and maladaptive risk-taking by promoting rigidity and impaired updating of legal appraisals.

In sum, this study addresses a knowledge gap by (a) distinguishing abstract and concrete legal cognition within a developmental and theoretical framework, and (b) separating NFC into distinct mechanisms to clarify its moderating role. This approach allows us to reconcile prior mixed findings and understand how these constructs interact to influence maladaptive risk-taking in adolescents and young adults.

## 2 Methods

### 2.1 Participants

Data were collected in March 2025 via Questionnaire Star during scheduled class sessions at two large public universities in eastern China. Of 583 returned questionnaires, 396 remained after data-quality screening. The final sample comprised 209 men (52.8%) and 187 women (47.2%), ages 17–25 (M = 21.53, SD = 1.87); majors were natural sciences (*n* = 194), humanities (*n* = 141), engineering (*n* = 54), and other (*n* = 7). Parental education was recorded as junior high or below (*n* = 112), high school/vocational (*n* = 143), and college or higher (*n* = 141). Informed consent was obtained from all participants (and from guardians where required).

Exclusions and rationale: Responses were removed if they (a) failed embedded attention checks, (b) had implausibly short completion times (< one-third of the pilot mean), or (c) displayed an identical response pattern (same response option on >90% of consecutive items). These criteria identify low-quality responding because extreme uniformity and very rapid completion rarely reflect valid, differentiated responses across heterogeneous constructs and typically coincide with failed attention checks.

Sampling and generalizability: We used convenience sampling of undergraduates enrolled in general-education courses (sampling frame: students at the two participating universities; theoretical population: Chinese university students). To reduce coverage bias we sampled across multiple faculties and course sections and administered the survey in class. Nonetheless, generalization to other cultures, regions, age groups, or non-student populations is limited.

### 2.2 Measures

*College Students’ Legal Cognition Assessment Scale.* We measured legal cognition using the College Students’ Legal Cognition Assessment Scale developed for Chinese college students (Xu and Yan, 2022). The scale comprises two subscales: concrete legal cognition (13 items; e.g., “The law guarantees citizens’ freedom of religious belief”) and abstract legal cognition (16 items; e.g., “Law is formulated by the state”). Items are rated on a 5-point Likert scale (1 = strongly disagree to 5 = strongly agree); higher scores indicate stronger legal cognition. The instrument was originally developed and validated for Chinese undergraduate populations to assess both knowledge- and value-oriented aspects of legal cognition. Prior validation reported acceptable structural validity and internal consistency in college samples (Xu and Yan, 2022). In the present sample, internal consistency was high: Cronbach’s *α* = 0.91 for the concrete subscale and α = 0.92 for the abstract subscale.

*The Need for Cognitive Closure Scale (NFCS)*. We administered the 21-item Chinese revision of the NFCS (Webster and Kruglanski, 1994; Chinese adaptation: Liu and Liang, 2007), which preserves two theoretically and empirically supported dimensions: need for structure (14 items) and decisiveness (7 items). Respondents rated items on a 6-point Likert scale (1 = strongly disagree, 6 = strongly agree); items 3, 14, 15, 17, 19, 20 and 21 were reverse scored. Subscale scores were computed as the sum of relevant items (higher scores = stronger trait expression). A sample item is “I do not like routine aspects of work or study.” In the current university sample, internal consistency was acceptable (need for structure α = 0.81; decisiveness α = 0.88; full scale α = 0.93).

Rationale and construct validity. The 21-item Chinese version was chosen because it is a validated, culturally adapted short form that retains the NFCS’s core processes: seizing (decisiveness—rapid closure) and freezing (need for structure — resistance to ambiguity). Individuals with low NFCS scores are comparatively tolerant of uncertainty: they seek additional information, consider alternative interpretations, and delay final judgments rather than rapidly seizing on a single conclusion.

*The Adolescent Risk-Taking Behaviors Questionnaire (ARQ)*. The Adolescent Risk-Taking Behaviors Questionnaire (ARQ–RB; Gullone et al., 2000), as revised and validated for Chinese adolescents by Zhang and Zhang (2016), was used to assess maladaptive risk-taking. The Chinese revision established measurement invariance across regional samples and demonstrated good structural validity and cross-group stability. The 17-item scale comprises four dimensions: sensation seeking (items 1–5), recklessness (items 10, 13), rebelliousness (items 6–12), and antisocial behavior (items 14–17). Consistent with the theoretical definition of maladaptive risk-taking, only the recklessness, rebelliousness, and antisocial subscales (12 items) were used in the present study. Responses were rated on a 5-point scale (0 = never to 4 = always), and summed to produce a total maladaptive risk-taking score, with higher scores indicating stronger tendencies toward maladaptive risk-taking. The subscale showed good internal consistency in this study (Cronbach’s *α* = 0.95).

### 2.3 Statistical analysis

Descriptive statistics and Pearson correlation analyses were conducted using SPSS 22.0 to examine the basic relationships among variables. To test for common method bias, Harman’s single-factor test was performed. Moderation effects were analyzed using Hayes’ PROCESS macro version 4.1 (Model 1) in SPSS. Specifically, the independent variables, moderator variables, and their interaction terms were entered into the model to examine the moderating role of the need for cognitive closure on the relationship between legal cognition and the outcome variables. All continuous variables were mean-centered before analysis to reduce multicollinearity. Statistical significance was set at *p <* 0.05.

## 3 Results

### 3.1 Common method bias test

To address potential common method bias, this study employed both procedural and statistical remedies. During data collection, procedural controls such as anonymous responses and reverse-coded items were implemented to reduce respondents’ evaluation apprehension and response tendencies. Statistically, the unmeasured latent method factor approach proposed by Podsakoff et al. (2003) was applied. The results indicated that adding the common method factor did not lead to a significant improvement in model fit (ΔCFI = 0.012, ΔTLI = 0.010, ΔRMSEA = 0.002). The variance explained by the method factor was 18.3%, well below the 50% threshold, and all item loadings on the method factor were nonsignificant (*p* > 0.05). These findings suggest that common method bias was not a serious concern in this study.

### 3.2 Descriptive statistics and correlation analysis

Observed score ranges and interquartile values were: abstract legal cognition 16–80(Q1–Q3 = 71–80), concrete legal cognition 13–65(Q1–Q3 = 57.5–65), need-for-structure 42.00–77.18 (Q1–Q3 = 59–71), decisiveness 12.82–39.18 (Q1–Q3 = 18–24), and maladaptive risk-taking 0–48 (Q1–Q3 = 2–27.5). Means (SD) were: concrete legal cognition M = 60.05 (SD = 6.87), abstract legal cognition M = 73.50 (SD = 8.36), need-for-structure M = 64.10 (SD = 8.57), decisiveness M = 22.18 (SD = 5.67), and maladaptive risk-taking M = 14.32 (SD = 15.56).

Univariate normality was assessed using skewness and kurtosis. Skewness (absolute values) ranged from 0.636 to 1.496 and kurtosis ranged from 0.16 to 1.675, all within conventional thresholds (*|*skew*|* < 2; *|*kurtosis*|* < 7), supporting the use of parametric tests.

Pearson correlations indicated that concrete and abstract legal cognition were each positively associated with need-for-structure (*r* = 0.35, *p* < 0.001; *r* = 0.36, *p* < 0.001, respectively) and negatively associated with maladaptive risk-taking (*r* = −0.30, *p* < 0.001; *r* = −0.27, *p* < 0.001, respectively). Neither concrete nor abstract legal cognition correlated significantly with decisiveness (*r* = −0.02, *p* > 0.05 for both). Need-for-structure was not significantly related to maladaptive risk-taking (*r* = 0.05, *p* > 0.05). Decisiveness was negatively associated with maladaptive risk-taking (*r* = −0.26, *p* < 0.001). Need-for-structure and decisiveness were negatively correlated (*r* = −0.25, *p* < 0.001).

### 3.3 Moderating effect of need for cognitive closure

To examine whether dimensions of the Need for Cognitive Closure moderate the relationships between legal cognition and maladaptive risk-taking, we estimated four hierarchical regression models (see Table 1). Model 1 tested the moderating role of need-for-structure on the link between concrete legal cognition and maladaptive risk-taking; Model 2 tested the same moderator for abstract legal cognition; Model 3 examined the main effect of decisiveness; and Model 4 tested decisiveness as a moderator of the relation between concrete legal cognition and maladaptive risk-taking. All analyses used PROCESS Model 1; predictors were standardized prior to analysis. Gender, age, and parental education were entered as covariates.

**Table 1:** The moderating effects of each dimension of need for cognitive closure on the relationship between legal cognition dimensions and maladaptive risk-taking behaviors.

Variable	Model1 (*β/t*)	Model 2 (*β/t*)	Model 3 (*β/t*)	Model 4 (*β/t*)
Constant	−0.31 (−2.50)*	−0.37 (−2.96)**	−0.44 (−3.65)***	−0.42 (−3.53)***
Gender	0.26 (3.23)**	0.29 (3.57)***	0.26 (3.30)**	0.25 (3.18)**
Age	−0.00 (−0.75)(*n.s.*)	−0.00 (−0.52)(*n.s.*)	−0.00 (−0.64)(*n.s.*)	−0.00 (−0.72)(*n.s.*)
PE	0.11 (2.22)*	0.12 (2.43)*	0.15 (3.11)**	0.14 (3.06)**
CLC	−0.45(−8.71)***			−0.31(−7.60)***
ALC		−0.38(−7.51)***		
NFC	0.15 (3.54)***	0.13 (3.01)**		
Decisiveness			−0.29(−6.93)***	−0.30(−7.09)***
CLC*NFC	−0.11(−4.34)***			
ALC*NFC		−0.08(−3.36)**		
CLC*Decisiveness				0.12 (2.31)*
*R^2^*	0.03***	0.02**	0.01*	0.01*
*F*	18.85***	11.29**	4.26*	5.33*

n.s., not significant (*p* > 0 0.05), ^*^p < 0.05, ^**^p < 0.01, ^***^p < 0.001. PE, parental education; CLC, concrete legal cognition; ALC, abstract legal cognition; NFC, need-for-structure.

Controlling for covariates, both concrete and abstract legal cognition negatively predicted maladaptive risk-taking. In Models 1 and 2, the interaction terms of concrete legal cognition × need-for-structure (*β* = −0.11, *t* = −4.34, *p* < 0.001) and abstract legal cognition × need-for-structure (*β* = −0.08, *t* = −3.36, *p* < 0.01) were significant and negatively associated with maladaptive risk-taking.

**Figure 1:**
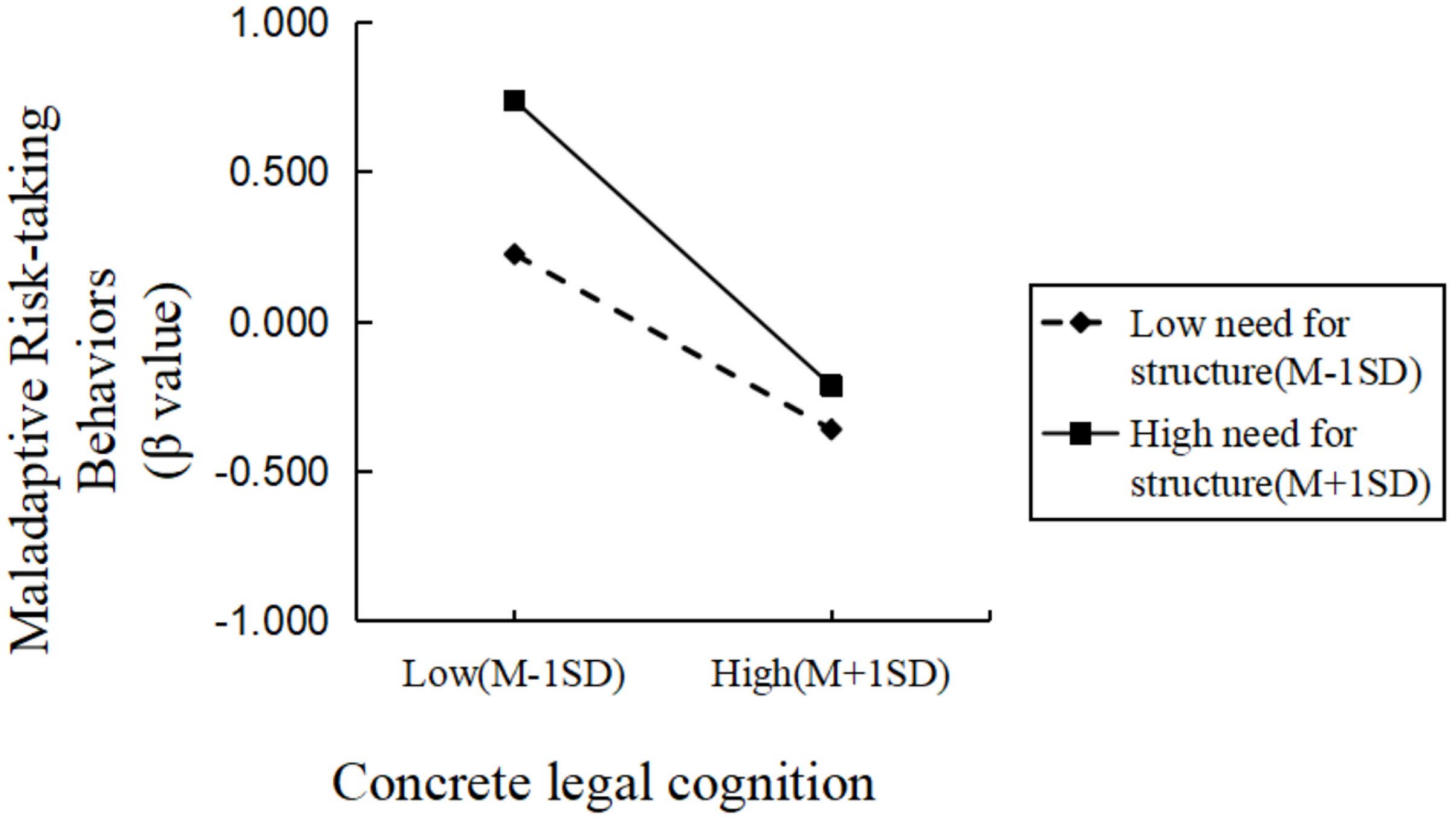
The moderating effect of need-for-structure on the relationship between concrete legal cognition and maladaptive risk-taking behaviors.

**Figure 2:**
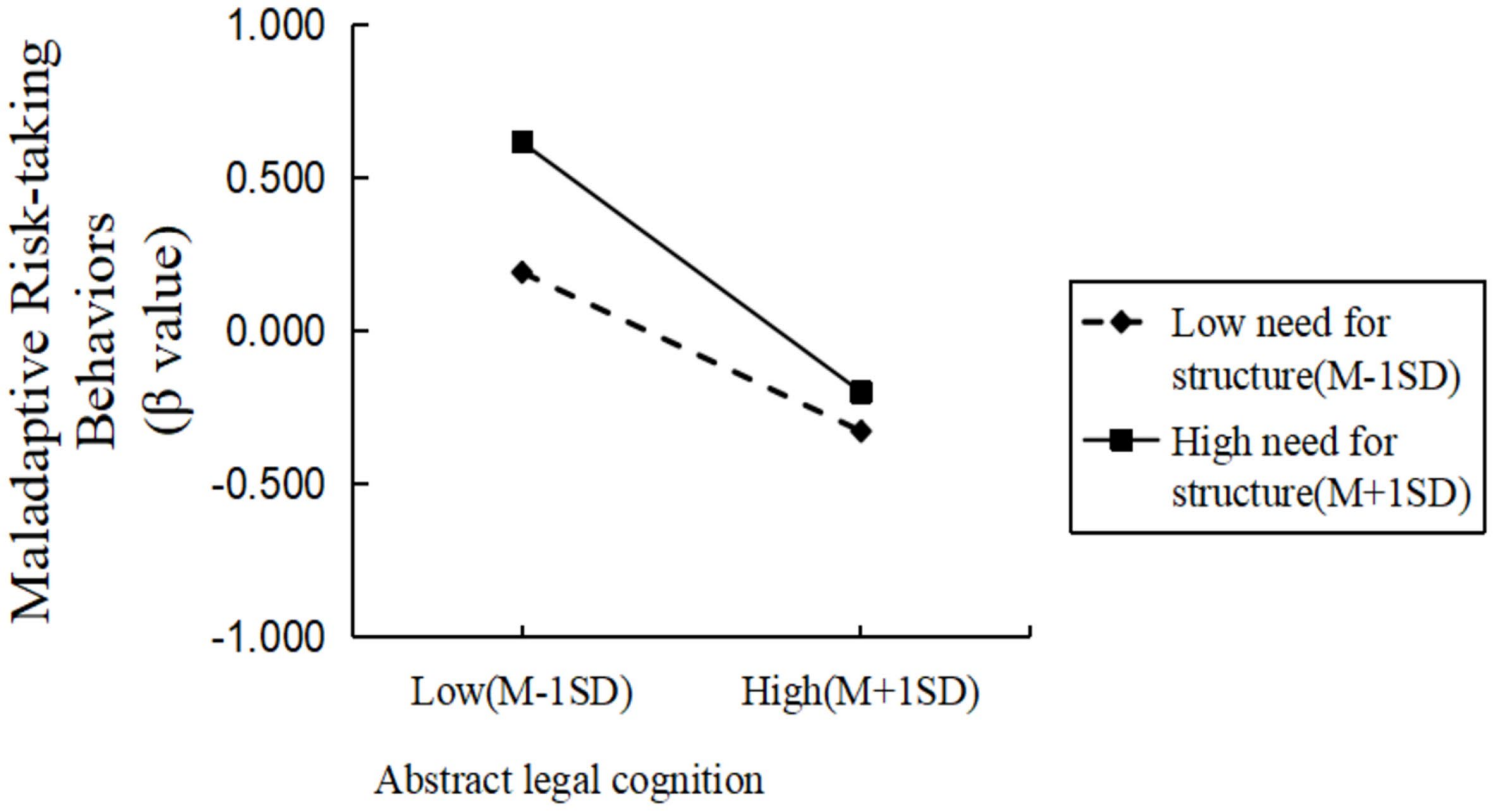
The moderating effect of structural need-for-structure on the relationship between abstract legal cognition and maladaptive risk-taking behaviors.

Simple-slope analyses, evaluated at one standard deviation above and below the mean of need-for-structure, indicated that the negative association between concrete legal cognition and maladaptive risk-taking was significant and stronger at high need-for-structure (βsimple = −0.56, *t* = −8.10, *p* < 0.001) than at low need-for-structure (βsimple = −0.34, *t* = −8.02, *p* < 0.001). Similarly, for abstract legal cognition the association was stronger at high need-for-structure (*β*simple = −0.46, *t* = −6.90, *p* < 0.001) than at low need-for-structure (βsimple = −0.29, *t* = −6.86, *p* < 0.001; see Figures 1, 2).

**Figure 3:**
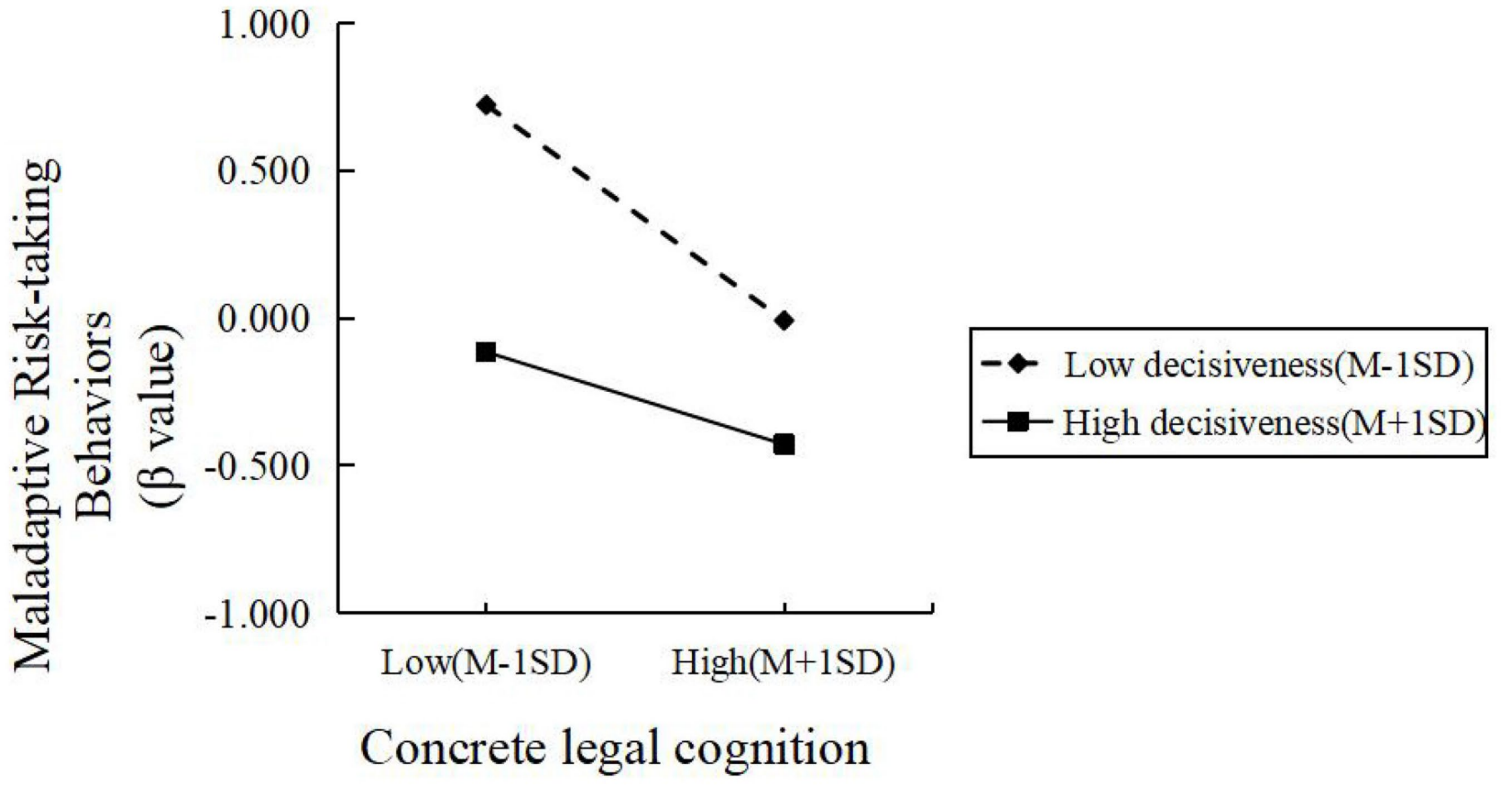
The moderating effect of structural need-for-structure on the relationship between abstract legal cognition and maladaptive risk-taking behaviors.

In Model 4, the interaction between concrete legal cognition and decisiveness was significant and positively associated with maladaptive risk-taking (*β* = 0.12, *t* = 2.31, *p* < 0.05), whereas the abstract legal cognition × decisiveness interaction was not significant (*β* = 0.08, *t* = 1.55, *p* > 0.05). Simple-slope tests showed that the protective effect of concrete legal cognition on maladaptive risk-taking was stronger at low decisiveness (*β*simple = −0.43, *t* = −5.64, *p* < 0.001) and attenuated at high decisiveness (*β*simple = −0.18, *t* = −3.31, *p* < 0.01), indicating that decisiveness weakens the protective effect of concrete legal cognition (see Figure 3).

In summary, the need-for-structure moderated the relationship between concrete legal cognition, abstract legal cognition, and maladaptive risk-taking behaviors. Specifically, as the level of need-for-structure increased, the negative predictive effect of concrete legal cognition and abstract legal cognition on maladaptive risk-taking behaviors strengthened. Decisiveness moderated the relationship between concrete legal cognition and maladaptive risk-taking behaviors. Specifically, as the level of decisiveness increased, the negative predictive effect of concrete legal cognition on maladaptive risk-taking behaviors weakened. That is, the moderating variable decisiveness attenuated the negative predictive effect of concrete legal cognition on maladaptive risk-taking behaviors. The moderating effect of the decisiveness dimension on the relationship between abstract legal cognition and maladaptive risk-taking behaviors were not significant.

## 4 Discussion

The present study found that both concrete and abstract legal cognition were negatively associated with maladaptive risk-taking among Chinese young adults, thereby supporting H1. These findings should be interpreted with caution given the cultural and sample context. The study was conducted among Chinese college students (average age ≈ 21), whose legal education, exposure to authority, and trust in institutions may differ from those in other cultural settings. Nevertheless, this pattern positions legal cognition as a proximal protective component of legal socialization, in line with prior research linking perceptions of legal legitimacy to lower levels of offending (Penner et al., 2014; Tankebe et al., 2016), and extends that literature by differentiating knowledge-based (concrete) and value-based (abstract) facets of legal cognition.

Critically, the need-for-structure appeared to strengthen the inhibitory association between both concrete and abstract legal cognition and maladaptive risk-taking (H2a supported). This moderation is consistent with the view that high NFC fosters rapid schema construction and intolerance of uncertainty, making individuals more likely to rely on legal knowledge and principles to avoid ambiguous, potentially harmful outcomes (Carleton et al., 2007; Schumpe et al., 2017; Webster and Kruglanski, 2014). In practical terms, those high in need-for-structure seem more apt to draw on principled (abstract) or rule-based (concrete) legal representations when evaluating risky options.

By contrast, decisiveness *weakened the association* between concrete legal cognition and maladaptive risk-taking, whereas it did not moderate the effect of abstract legal cognition (H2b partially supported). This pattern *suggests* that decisiveness promotes cognitive rigidity and premature closure on heuristic judgments, *which may u*ndermine the application of specific legal contingencies in contexts requiring flexible or updated appraisal (Berenbaum, 2010; Disatnik and Steinhart, 2015; Jackson, 2015). The absence of a similar effect for abstract legal cognition *might reflect* the higher-order, principle-driven nature of abstract legal beliefs, which are less vulnerable to dispositional rigidity and thus tend to remain associated with lower risk-taking even when decisiveness is high (Giacomantonio et al., 2014; Zara and Farrington, 2019).

These results should be considered within the cultural boundaries of the sample. Legal socialization and attitudes toward law can be culture-specific; for instance, Chinese college students may possess different baseline levels of legal knowledge or institutional trust compared to those in other countries, potentially influencing both their legal cognition and risk-related behavior. Future research should replicate these findings in more diverse populations—including non-student youth, working young adults, and cross-cultural samples—to test their generalizability.

Theoretically, the results refine accounts of compliance by indicating that motivational closure processes (need-for-structure vs. decisiveness) differentially relate to the behavioral influence of distinct legal-cognitive representations, thereby linking cognitive-motivational and legal-socialization perspectives (Kruglanski et al., 1993; Roets and Van Hiel, 2011). Practically, interventions that aim to enhance legal knowledge alone may not be sufficient for individuals high in decisiveness; programs should also focus on increasing tolerance for uncertainty and fostering reflective, flexible decision-making (Webster and Kruglanski, 2014; Carleton et al., 2007). Supporting cognitive resources (e.g., working memory) could further help individuals apply legal knowledge effectively under pressure (Webster and Kruglanski, 2014).

Finally, these patterns provide preliminary insights into broader conceptions of the legal subject: the differential interaction between decisiveness and legal cognition may mirror tensions among biological, rational, and social dimensions of identity, whereby heightened decisiveness privileges more immediate, biologically anchored tendencies over deliberative, rationalized legal reasoning (Liu, 2024; Cao, 2006). Overall, this study suggests associations rather than causal effects, clarifying how distinct NFC processes may shape the protective role of legal cognition and highlighting potential cognitive–motivational targets for reducing maladaptive risk-taking.

## 5 Conclusion and implications for adolescent crime prevention

This study found that both concrete and abstract legal cognition were linked to lower maladaptive risk-taking among Chinese college students, while Need for Cognitive Closure (NFC) dimensions appeared to moderate these associations: the need-for-structure strengthened the protective relation of legal cognition, whereas decisiveness weakened the association between concrete legal knowledge and lower risk-taking. The pattern implies that an excessive preference for certainty may undermine the flexible, deliberative application of legal information, even as a stronger need-for-structure seems to promote the mobilization of both principled and rule-based legal representations.

Practically, these findings suggest integrated, profile-sensitive prevention strategies (Steinberg, 2008; Farrington, 1989). First, curricula and community programs should strengthen both concrete (e.g., statutes, sanctions, cases) and abstract (e.g., justice, rights, legitimacy) legal cognition to foster law-abiding orientations. Second, for adolescents high in decisiveness, pairing legal education with activities that promote uncertainty tolerance and reflective decision-making—such as “what-if” simulations or perspective-taking exercises—may reduce premature closure and encourage flexible use of legal knowledge (Carleton et al., 2007; Webster and Kruglanski, 2014). Third, interventions should leverage family, school, and media contexts to tailor messaging: principle-focused narratives may resonate more with youths motivated by order, whereas case-based, consequence-focused stories could better engage those less reflective (Baumert and Schmitt, 2016).

Several methodological limitations merit note. Reliance on self-report measures may introduce social-desirability and common-method bias (Podsakoff et al., 2003); convenience sampling of Chinese university students restricts external validity across regions, age cohorts, and non-student populations; the cross-sectional design precludes temporal and causal inference; and use of revised (rather than original) instruments—despite acceptable internal consistency—may compromise measurement validity. Future research should employ longitudinal or experimental designs, recruit more diverse and representative samples, and adopt multi-method assessment (e.g., behavioral indicators, informant/administrative records) alongside rigorous scale validation to bolster causal, measurement, and generalizability claims.

In sum, aligning legal education with individual differences in cognitive closure represents a promising, yet preliminary, approach to reducing early maladaptive risk-taking. Future longitudinal and experimental studies should test whether such mechanisms causally predict delinquency or rule-breaking behavior.

## Data Availability

The original contributions presented in the study are included in the article/Supplementary material, further inquiries can be directed to the corresponding authors.
