# Correction: The relationship between college students' legal cognition and maladaptive risk-taking behaviors: the moderating effect of need for cognitive closure

**DOI:** 10.3389/fpsyg.2025.1756037

**Published:** 2025-12-15

**Authors:** Yu Hu, Qiang Ding, Hui Liu, Shuhui Xu

**Affiliations:** 1 Department of Psychology, Wenzhou University, Wenzhou, Zhejiang, China; 2 Center for Psychology and Behavior Research, Wenzhou University, Wenzhou, Zhejiang, China; 3 College of International Education, Wenzhou University, Wenzhou, Zhejiang, China; 4 School of Foreign Languages, Northwest University, Xi'an, Shaanxi, China

**Keywords:** legal cognition, concrete legal cognition, abstract legal cognition, need for cognitive closure, need for structure, decisiveness

There was a mistake in Figure 3 as published. Figure 3 was erroneously duplicated from Figure 2.

The corrected Figure 3 and its caption appear below.

**Figure 3:**
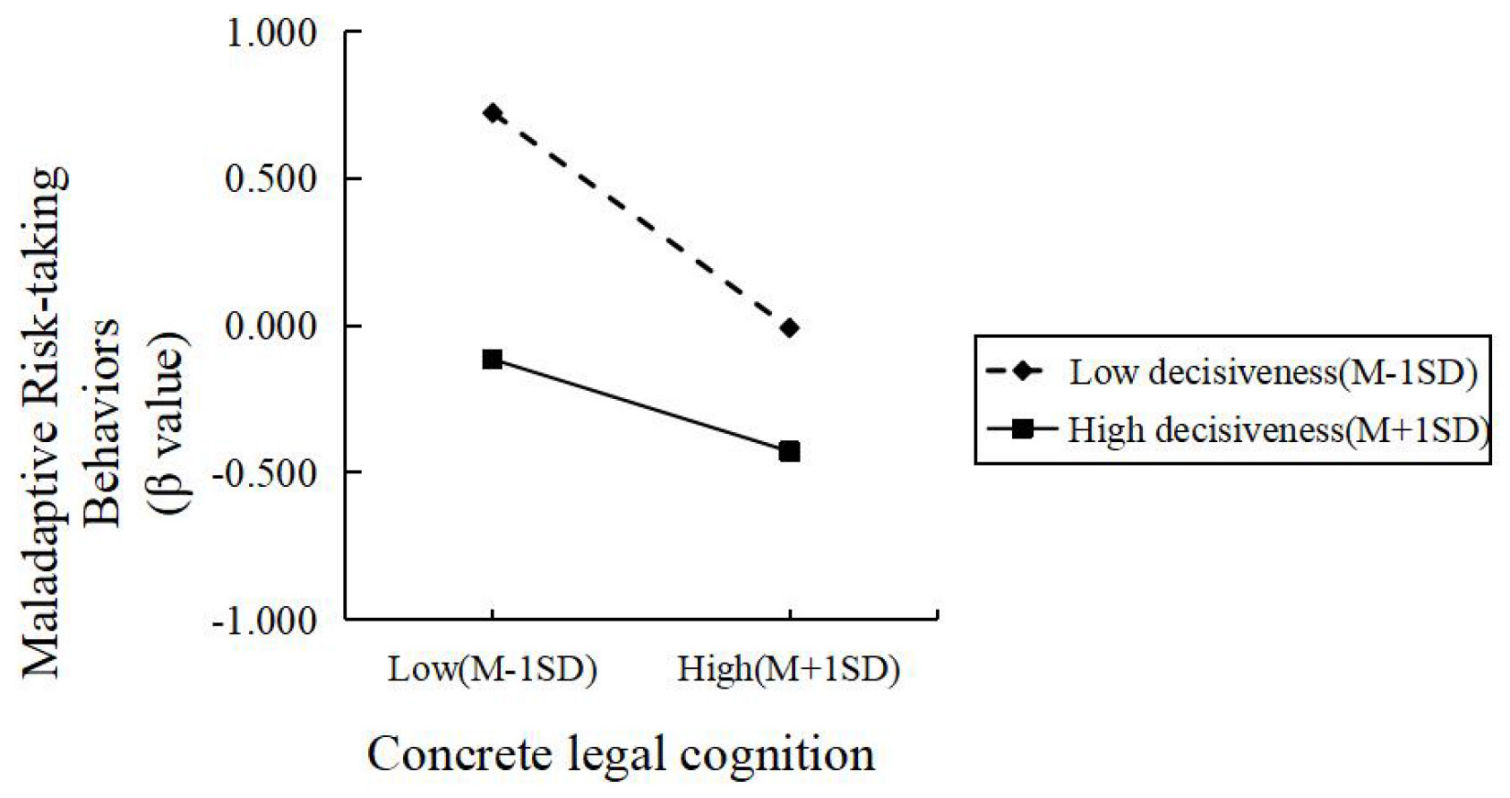
The moderating effect of decisiveness on the relationship between concrete legal cognition and maladaptive risk-taking behaviors.

The original version of this article has been updated.

