# Supplementary material for: The relationship between college students’ legal cognition and maladaptive risk-taking behaviors: the moderating effect of need for cognitive closure
Source: Front Psychol. 2025 Nov 26;16:1717060. doi: 10.3389/fpsyg.2025.1717060 (PMC12690936; doi:10.3389/fpsyg.2025.1717060)
Supplement: Supplementary file 1 [file Supplementary_file_1.docx]

**Appendix I: Need for Cognitive Closure Scale**

Dear Student,

Hello!

This assessment pertains to cognitive styles and risk-taking behaviors among university students. Please select the options that best reflect your own situation. There are no right or wrong answers; respond based on your intuition without any reservations. We kindly ask you to patiently complete this questionnaire. Your participation is sincerely appreciated!

Below are a series of statements related to law. Please rate the extent to which each statement aligns with your personal views using a scale of 1 to 5.

The numbers 1–5 represent:

1 – Strongly Disagree

2 – Somewhat Disagree

3 – Uncertain

4 – Somewhat Agree

5 – Strongly Agree

1. I do not like uncertain situations.
2. I do not like questions that could have many different answers.
3. I like having friends whose behavior is unpredictable.*
4. I find that an orderly, regular lifestyle suits my personality.
5. When eating out, I prefer to go to places I have been to before because I know what to expect.
6. I hate having to change my plans at the last minute.
7. I believe that being organized and disciplined is one of the most important characteristics of a good student.
8. I am reluctant to spend time with people who might behave in unexpected ways.
9. I enjoy socializing with familiar friends because I know their habits.
10. I find that establishing consistent routines helps me enjoy life more.
11. I prefer a well-organized way of life.
12. I like to have a place for everything and to keep everything in its place.
13. I feel uncomfortable when I am unsure about someone's meaning or intentions.
14. I do not like the routine aspects of my work or studies.*
15. When shopping, I find it hard to decide what I really want.*
16. When facing a problem, I can usually quickly identify the best solution.
17. I tend to put off making important decisions until the last moment.*
18. I usually make important decisions quickly and confidently.
19. I consider myself an indecisive person.*
20. I often hesitate when making most decisions.*
21. When trying to solve a problem, I often feel overwhelmed by seeing too many possible options.*

**Appendix II: University Students' Legal Cognition Assessment Scale**

The following are statements regarding the need for cognitive closure. Please rate the extent to which each statement aligns with your views using a scale of 1 to 6.

The numbers 1–6 represent:

1 – Strongly Disagree

2 – Moderately Disagree

3 – Slightly Disagree

4 – Slightly Agree

5 – Moderately Agree

6 – Strongly Agree

1. I believe that laws are established by the state.
2. I believe that laws are enforced through state power.
3. I believe that socialist law in China reflects the will of the people.
4. I know that the "Constitution of the People's Republic of China" is the fundamental law of the country.
5. I know that the "Constitution of the People's Republic of China" holds the highest legal authority.
6. I believe that no organization or individual may enjoy privileges beyond the Constitution and the law.
7. I know that China's Constitution stipulates that the system of people's congresses is the fundamental political system.
8. I believe that all citizens of the People's Republic of China are equal before the law.
9. I believe that the rights and interests of the people must be safeguarded by the law.
10. I believe that the authority of the law must be upheld by the people.
11. I believe in the integration of rule of law and rule of virtue.
12. We must firmly establish the concept of the supremacy of the Constitution and the law.
13. We must cultivate the basic concept of equality before the law for everyone.
14. I believe that respecting and safeguarding the authority of the law is crucial for the comprehensive advancement of the rule of law.
15. I believe that assisting vulnerable groups is stipulated and protected by Chinese law.
16. I believe that citizens' lawful private property is inviolable.
17. I believe that the law should protect my personal privacy rights.
18. I know that the law does not permit the unlawful deprivation of a citizen's right to life.
19. I know that citizens have the right to vote in accordance with the law.
20. I believe that citizens have the right to equal employment opportunities.
21. I believe that the law guarantees citizens' freedom of religious belief.
22. I know that citizens have the right to apply to attend public trials conducted by the People's Courts.
23. I know that it is not permitted to disclose someone else's private information without their consent.
24. I believe that parents have the duty to raise and educate their minor children.
25. I believe that adult children have the duty to support and assist their parents.
26. I believe that citizens of the People's Republic of China have the duty to pay taxes in accordance with the law.
27. I know that safeguarding national sovereignty and territorial integrity is a common obligation of all Chinese people.
28. I believe that legal rights and legal obligations are interdependent and inseparable.
29. I believe that legal rights and legal obligations are equal.

**Appendix III: Adolescent Risk Questionnaire – Risk Behavior Scale**

The following are descriptions of risk behaviors. Please rate your actual level of involvement in these behaviors in your daily life using a scale of 0 to 4.

The numbers 0–4 represent:

0 – Never

1 – Rarely

2 – Sometimes

3 – Often

4 – Always

1. Skiing
2. Practicing taekwondo, martial arts, or boxing
3. Rollerblading
4. Skydiving
5. Signing up for activities (e.g., competitions, performances) organized by the school or community
6. Skipping school
7. Drinking alcohol while underage
8. Smoking
9. Getting drunk
10. Using drugs
11. Staying out very late
12. Riding a bicycle after drinking alcohol
13. Engaging in unsafe sexual behavior
14. Overeating
15. Teasing and bullying others
16. Cheating
17. Starting conversations with strangers
