# Supplementary material for: The relationship between college students’ legal cognition and maladaptive risk-taking behaviors: the moderating effect of need for cognitive closure
Source: Front Psychol. 2025 Nov 26;16:1717060. doi: 10.3389/fpsyg.2025.1717060 (PMC12690936; doi:10.3389/fpsyg.2025.1717060)
Supplement: Supplementary file 2 [file Supplementary_file_2.docx]

****Appendix B****

****Item-Total Correlation Results for Measurement Scales****

****Table 1****
Item-Total Correlations for the Legal Cognition Scale (N = 396)

| **Item** | **Item-Total Correlation** | **Item** | **Item-Total Correlation** | **Item** | **Item-Total Correlation** |
| --- | --- | --- | --- | --- | --- |
| l28 | 0.779 | l10 | 0.668 | l25 | 0.620 |
| l6 | 0.756 | l8 | 0.667 | l13 | 0.615 |
| l12 | 0.749 | l2 | 0.666 | l15 | 0.520 |
| l9 | 0.746 | l26 | 0.666 | L29 | 0.436 |
| l17 | 0.744 | l11 | 0.645 | l1 | 0.385 |
| l18 | 0.734 | l22 | 0.644 |  |  |
| l24 | 0.733 | l21 | 0.643 |  |  |
| l5 | 0.732 | l3 | 0.623 |  |  |
| l14 | 0.725 | l7 | 0.623 |  |  |
| l16 | 0.721 |  |  |  |  |

Note: The scale's overall Cronbach's α = 0.957.

****Table 2****
Item-Total Correlations for the Risk-Taking Behavior Scale (N = 396)

| Item | Item-Total Correlation | Item | Item-Total Correlation |
| --- | --- | --- | --- |
| r12 | 0.914 | r7 | 0.813 |
| r8 | 0.900 | r17 | 0.787 |
| r10 | 0.888 | r11 | 0.771 |
| r9 | 0.879 | r6 | 0.700 |
| r13 | 0.873 | r14 | 0.676 |
| r15 | 0.871 |  |  |
| r16 | 0.864 |  |  |

Note: The scale's overall Cronbach's α = 0.959.

****Table 3****
Item-Total Correlations for the Need for Cognitive Closure Scale (N = 396)

| Item | Item-Total Correlation | Reverse-Scored |
| --- | --- | --- |
| n4 | 0.714 | No |
| n14 | 0.713 | Yes |
| n8 | 0.701 | No |
| n12 | 0.690 | No |
| n17 | 0.690 | Yes |
| n15 | 0.684 | Yes |
| n5 | 0.683 | No |
| n13 | 0.681 | No |
| n2 | 0.680 | No |
| n16 | 0.674 | No |
| n10 | 0.667 | No |
| n11 | 0.663 | No |
| n20 | 0.658 | Yes |
| n3 | 0.648 | Yes |
| n21 | 0.645 | Yes |
| n9 | 0.631 | No |
| n19 | 0.627 | Yes |
| n6 | 0.588 | No |
| n7 | 0.579 | No |
| n1 | 0.578 | No |
| n18 | 0.577 | No |

Note: The scale's overall Cronbach's α = 0.932. Reverse-scored items: n3, n14, n15, n17, n19, n20, n21.

****Summary of Results****

****Legal Cognition Scale:**** All items demonstrated item-total correlations above the acceptable threshold of 0.30, with 29 items exceeding 0.50. This indicates excellent internal consistency for the scale.

****Risk-Taking Behavior Scale:**** All items demonstrated item-total correlations above 0.60, with the majority exceeding 0.80. This indicates exceptionally high internal consistency for the scale.

****Need for Cognitive Closure Scale:**** All items demonstrated acceptable item-total correlations (>0.577). The scale exhibited excellent overall internal consistency (α = 0.932). Reverse-scored items performed notably well, with several among the top-ranked items.

****Reliability Indicators:**** The Cronbach's alpha coefficients for all three scales were above 0.93, indicating excellent reliability levels.

These results support the strong measurement reliability of all three scales within the context of the present study, confirming their suitability for subsequent data analysis.
